# Looking at the Possibility of Using Mushroom Mycelium for Developing Leather-like Materials Aligned with Eco-Friendly and Sustainable Fashion Trends

**DOI:** 10.3390/life15111746

**Published:** 2025-11-13

**Authors:** Worawoot Aiduang, Thanawin Patipattanakul, Yutthaphum Keduk, Apiwit Rattanapat, Phumin Phumila, Praween Jinanukul, Phongeun Sysouphanthong, Orlavanh Xayyavong, Kritsana Jatuwong, Saisamorn Lumyong

**Affiliations:** 1 Office of Research Administration, Chiang Mai University, Chiang Mai 50200, Thailand; worawoot.aiduang@cmu.ac.th (W.A.); kritsana.ja@cmu.ac.th (K.J.); 2 Department of Biology, Faculty of Science, Chiang Mai University, Chiang Mai 50200, Thailand; psphongeun@gmail.com (P.S.); xorlavanh@yahoo.com (O.X.); 3 Secondary School, Montfort College, Muang Chiang Mai, Chiang Mai 50000, Thailand; thanawin4488@gmail.com (T.P.); Poomkedook@gmail.com (Y.K.); apiwitrattanapat@gmail.com (A.R.); din25499@gmail.com (P.P.); 4 Faculty of Architecture, Chiang Mai University, Chiang Mai 50200, Thailand; praween_ji@cmu.ac.th; 5 Department of Biology, Faculty of Science, Champasack University, Pakse 16010, Laos; 6 Center of Excellence in Microbial Diversity and Sustainable Utilization, Chiang Mai University, Chiang Mai 50200, Thailand

**Keywords:** biomass utilization, eco-friendly materials, innovative design, mycelium technology, SDGs 8 and 9

## Abstract

The growing demand for sustainable alternatives to animal and synthetic leathers has accelerated interest in mycelium-based materials as an eco-friendly solution for the fashion industry. This study explores the potential of mushroom mycelium to create leather-like materials that align with circular fashion principles. Five species of edible and medicinal mushrooms were cultivated on sawdust substrates and evaluated for their growth performance, physical properties, and suitability as leather substitutes. Growth analysis revealed distinct species-specific behaviors: *Cubamyces flavidus* and *Lentinus squarrosulus* exhibited rapid colonization, achieving full substrate coverage within five days and forming dense mycelial networks at 14 days. In contrast, despite growing more slowly, *Sanghuangporus vaninii* and *Ganoderma gibbosum* formed thicker, more compact mats that might be suitable for strong leather-like materials. Visual and structural assessments showed diverse textures, colors, and hyphal architectures resembling natural leather. Physical characterization revealed shrinkage ranging from 13.17% to 24.09%, higher than for cow tanned leather (>5%) and PU microfiber (0.1–1.2%), suggesting a need for stabilization treatments. Apparent densities ranged from 0.13 g/cm^3^ to 0.30 g/cm^3^, lower than those of cow leather (0.49 g/cm^3^) and PU leather (0.38 g/cm^3^), highlighting species-specific hyphal structures that influence flexibility, porosity, and strength. SEM imaging confirmed the presence of interwoven hyphal mats resembling the fibrous architecture of natural leather, with *S. vaninii* showing the most uniform and continuous structure. Water absorption was significantly higher in mycelium sheets, consistent with their microporous nature, though *S. vaninii* showed the lowest uptake, reflecting possible natural water absorption. Thermogravimetric analysis revealed three-stage degradation profiles, with *S. vaninii* and *G. gibbosum* retaining >35% mass at 400 °C, indicating strong thermal stability for processing techniques such as hot pressing and finishing. Overall, the results demonstrate mycelium-based leathers as a biodegradable, low-impact alternative that can replicate the visual and functional characteristics of traditional leather, with opportunities for further improvement in substrate optimization, eco-tanning, surface coating, and scalable production toward a sustainable fashion future.

## 1. Introduction

The growing need to address environmental degradation and reduce the ecological footprint of industries has driven innovation in sustainable material development [1]. Among these, the fashion industry, which has long been criticized for its environmental effect and resource-intensive methods, is aggressively looking at substitutes for conventional textiles, especially leather made from animals [2]. Globally, the leather industry is estimated to produce over 130 million tons of carbon dioxide emissions annually and generate approximately 25% of all chromium-containing waste released into the environment [3,4]. Traditional leather production not only relies heavily on livestock farming, which contributes greenhouse gas emissions, deforestation for raising cattle, high energy and water consumption, and waste management issues, but also involves tanning processes that utilize toxic chemicals such as chromium, posing serious risks to ecosystems and human health [5]. In response to these challenges, researchers and designers are turning toward bio-based, biodegradable, and cruelty-free materials that align with eco-friendly values and support the global movement toward circular economy models [6]. One such possible alternative is mycelium-like leather, a material derived from the vegetative root-like structure of fungi, which offers a sustainable and adaptable substitute for synthetic and animal-based leather [7].

Mycelium, the filamentous network of fungal hyphae, is well-known for its ability to grow rapidly on various lignocellulosic agricultural wastes, forming dense, fibrous mats that can be engineered into a range of shapes and textures. These unique properties make mycelium an attractive choice for use in the creation of leather-like materials that replicate the texture, flexibility, and appearance of animal leather [8]. The production process of mycelium-like leather (often referred to as “myco-leather”) involves cultivating various fungal strains under controlled conditions, followed by processing techniques such as drying and cross-linking treatment [9,10,11]. Mycelium-like leather is a feasible option for eco-conscious and sustainable fashion manufacturers together because it is renewable, biodegradable (over 90% degradation in natural environments within a few months), and requires less resources to produce than synthetic or animal-based alternatives [12,13].

This study explores the potential of mushroom mycelium from various fungal species to be developed into leather-like materials suitable for sustainable fashion applications, with the objective of obtaining viable alternatives to conventional leather. The research focuses on cultivating selected fungal species on rubber sawdust, an agro-industrial byproduct, to generate dense mycelial mats suitable for material testing. These mats were then tested through a number of primary physical and structural assessments. These properties provide information about the potential of mycelium-like leather to achieve the technical demands of fashion materials, including durability, ease, and appearance. The findings of this research not only demonstrate the viability of using mushroom mycelium as an alternative leather source but also additionally uncover important characteristics that influence its performance in various applications.

While the results are promising, there remain several challenges and opportunities for future development. Ensuring consistent quality, scalability of production, resistance to wear and tear, and consumer acceptance are critical steps before mycelium-like leather can compete with traditional materials in mainstream markets [7]. Additionally, surface coating, tanning, and combination layering techniques may be needed to enhance specific properties such as tensile strength and waterproofing [8,14,15,16]. Nonetheless, with growing consumer demand for ethical and sustainable alternatives, the integration of mycelium-like leather into the fashion industry presents an interesting pathway toward more responsible consumption and production patterns [17].

Thus, this study aims to link the knowledge divide between material science, biological innovation, and trends by providing a comprehensive examination of the structural and physical characteristics of leather-like materials made from mycelium in comparison to commonly used leather materials that include cow tanned leather and polyurethane microfiber leather. It also presents practical methods and ideas for simple, accessible production that link recently developed techniques for inducing mycelium-like leather with conventional methods reported in previous studies. Together, these investigations and methodological outcomes provide significant information on the properties of specific fungal species and the improvement of production processes. Furthermore, the study looks at future prospects, potential technological advancements, and key challenges that must be addressed to support the transition from experimental prototypes to sustainable, commercially viable products.

## 2. Materials and Methods

### 2.1. Source of Mushroom Mycelium and Inoculum Preparation

Five species of edible and medicinal mushroom mycelia were selected for this study based on their rapid growth and high potential for consistency in density, structure, and surface texture, which are key characteristics for producing efficient leather-like materials. These species were obtained from the culture collections of the Saisamorn Lumyoung Laboratory Culture Collection (SLLC), Faculty of Science, Chiang Mai University. The selected species included *C. flavidus* (CMU-AM011), *G. gibbosum* (CMU-WE017), *L. squarrosulus* (CMU-WE001), *P. similis* (CMU-AM006), and *S. vaninii* (CMU-WE058). Prior to use, each species was sub-cultured onto potato dextrose agar (PDA) plates and incubated at 30 °C for seven days in darkness to preserve active growth [18].

For inoculum preparation, sorghum grains were used as a nutrient-rich substrate. The grains were thoroughly washed and boiled for 20 min. After boiling, remaining water was carefully drained to prevent excess moisture during sterilization. A total of 150 g of the prepared grains were then put into glass bottles, sealed with cotton plugs, and sterilized by autoclaving at 121 °C for 20 min. Once cooled to room temperature, each bottle was inoculated with five 5 mm diameter mycelial plugs taken from the PDA cultures of the respective mushroom species. The inoculated bottles were then incubated at 30 °C for two weeks, allowing the mycelium to fully colonize the sorghum substrate [19].

### 2.2. Substrate Use and Preparation

Rubber tree sawdust was selected as the primary substrate for this study. The sawdust was sourced from a sawmill in northern Thailand. Prior to use, it was sieved to obtain particles between ≤2 mm in size and then oven-dried at 60 °C until completely moisture-free, as investigated by visual inspection and stable weight measurements [20].

### 2.3. Measurement of Mycelial Development on Sawdust

Sawdust was combined with the nutritional supplement for mushroom growing (5% rice bran, 1% calcium carbonate, 2% calcium sulfate, and 0.2% sodium sulfate). The mixtures were then adjusted to 60% relative humidity by adding water. A 10 g portion of each substrate mixture was placed onto a glass plate (90 mm in diameter) and sterilized at 121 °C for 60 min. After cooling, a 10 mm mycelial disk was placed at the center, and plates were incubated at 30 °C for 14 days [18]. Mycelial growth was measured daily by recording the colony diameter along two perpendicular axes, and the radial growth rate (mm/day) was calculated based on the increase in average colony diameter over time. Growth density was evaluated on day 7 and at the end of incubation, classified as very thin (+), thin (++), thick (+++), or very thick (++++) following the criteria described by Raman et al. [12]. Each treatment was conducted in five replicates.

### 2.4. Mushroom Mycelium Cultivation on Substrate

The dried substrate was mixed with the nutritional supplement (as described in Section 2.3) based on dry weight, and adjusted to a final moisture content of approximately 60% relative humidity. A total of 600 g of the prepared substrate was packed into polypropylene bags (3.5 × 12.5 in). Each bag was sealed using cotton-plugged polyvinyl chloride pipe rings and covered with paper. The bags were sterilized in an autoclave at 121 °C for 60 min. After sterilization, the bags were allowed to cool to room temperature over 24 h. Each bag was then inoculated with six grams of mycelial culture, achieving a substrate-to-inoculum ratio of 100:1 (*w*/*w*). The inoculated bags were incubated in darkness at 30 °C for 30 days, or until the mushroom mycelium fully colonized the substrate [21].

### 2.5. Induction of Mycelium-like Leather

**Figure 1:**
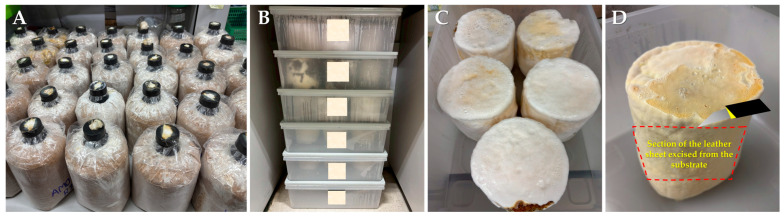
An overview of the primary processes involved in inducing mycelium-like leather in this study: (**A**) colonized spawn substrate, (**B**) incubation of the spawn, (**C**) development of dense mycelial mats, and (**D**) separation of the compact mats from the substrate.

Fully colonized substrate bags for each mushroom species were used to induce mycelium-like leather following modified methods from previous studies (Figure 1) [12,22,23]. The colonized spawn substrate was carefully removed from the bags and placed in polypropylene plastic boxes (38.8 × 28.3 × 14.2 mm^3^), then incubated in the dark at 28–30 °C (room temperature) with 80–90% humidity for 30 days to allow for further mycelial growth around the outer surfaces. After 30 days, dense mycelial mats were carefully separated from the substrate and cut into sheets measuring 10 × 20 cm^2^. Any residual substrate adhering to the sheets was carefully scraped off using a scalpel and cleaned with a soft brush. The fresh volume of each sheet was recorded to calculate average shrinkage. Sheets were then air-dried for 24 h, followed by oven-drying at a controlled temperature (70 °C) for 24–48 h until a constant weight was achieved [12]. The dried mycelial sheets were then stored in desiccators for further experiments.

### 2.6. Investigating the Physical Properties and Characteristics of Mycelium-like Leather

#### 2.6.1. Assessing Visual Characteristics

A preliminary visual assessment was carried out to examine the morphological features of the mycelium-like leather sheets after drying, following a modified protocol adapted from Crawford [23]. Comparative observations were conducted to evaluate overall appearance, with particular attention given to color and texture. Color characterization was recorded using the *Methuen Handbook of Colour* as a reference standard [24], focusing on the parts with the most surface area to achieve similarity. In addition to surface appearance, each sheet was systematically analyzed according to mushroom species, decay type, and hyphal system, based on the morphological characteristics of their fruiting bodies. The classification followed the guidelines provided in manuals of common edible and medicinal mushrooms, complemented by relevant academic literature [25,26,27,28]. These parameters provided a comprehensive dataset crucial for identifying the most suitable fungal species for high-quality mycelium-like leather production.

#### 2.6.2. Shrinkage and Density Measurements

**Figure 2:**
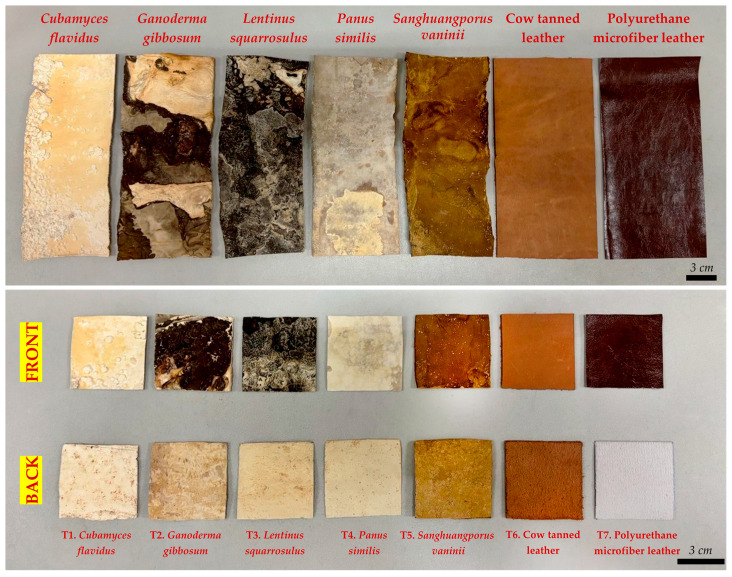
Comparative specifications of mycelium-like leather sheets versus conventional synthetic and animal-derived leather.

Shrinkage of the mycelium-like leather sheets was measured by comparing their wet and dry volumes, which were calculated from measurements of length, width, and thickness, following the modified method described by Pahlawan & Griyanitasari [29]. The shrinkage percentage was calculated using the formula: Shrinkage (%) = [(V_1_ − V_2_)/V_1_] × 100, where V_1_ represents the wet volume and V_2_ the dry volume of the sample. After shrinkage measurements, the dried mycelium-like leather sheets were cut into 5 × 5 cm^2^ squares. Density was determined using the dry mass and volume, following the method described by Raman et al. [12] and adapted to the International Organization for Standardization (ISO) 2420 [30]. Each sample was measured five times for certainty. The resulting differences in physical characteristics among the mycelium-like leather sheets are illustrated in Figure 2 for comparative analysis.

#### 2.6.3. Scanning Electron Microscope Observation

The surface structures of the mycelium-like leather sheets were examined using scanning electron microscopy (SEM JSM-IT300, JEOL, Tokyo, Japan). Dehydrated sheets were cut into small squares approximately 5 × 5 mm^2^ with a scalpel. These pieces were then mounted on 10 mm^2^ stub adapters using 2 mm^2^ double-sided carbon tape. The samples were coated with gold for two minutes under high vacuum conditions to enhance conductivity. SEM imaging was performed at a high voltage of 15 kV at the Science and Technology Service Center, Faculty of Science, Chiang Mai University, Thailand. Surface structures from each treatment group were analyzed by comparing the captured SEM images to identify differences [21].

#### 2.6.4. Water Absorption Testing

The surface hydrophobicity of the mycelium-like leather sheets was assessed by measuring water absorption, following a modified method from Zhang et al. [31]. The samples were cut into small pieces (approximately 5 × 5 cm) and weighed to obtain the initial mass (W_0_). Each piece was then submerged in a beaker containing an appropriate volume of water and removed at intervals of 30, 60, 90, 150, 210, 270, and 330 min. After removal, the surface water was gently blotted using filter paper, and the wet mass (W_t_) was recorded. The following formula was used to determine water absorption: Water absorption (%) = (W_t_ − W_0_)/W_0_ × 100. Each sample was tested five times, and the average value was reported.

#### 2.6.5. Thermogravimetric Analysis (TGA)

The thermal degradation behavior of mycelium-like leather sheets was analyzed using a thermogravimetric analyzer (Rigaku: Thermo Plus EVO2, Nihon Rigaku Co., Tokyo, Japan). Approximately 2.5–3.0 mg of each sample was placed in an alumina crucible and heated from 25 °C to 600 °C at a constant rate of 10 °C/min under a nitrogen atmosphere [12].

### 2.7. Statistical Analysis

Statistical analysis of the experiments was performed using one-way analysis of variance (ANOVA) with SPSS software, version 17.0 (SPSS Inc., Chicago, IL, USA). Duncan’s multiple range test was applied to determine significant differences between mean values at a significance level of *p* ≤ 0.05.

## 3. Results and Discussion

### 3.1. Mycelial Growth Characteristics

**Figure 3:**
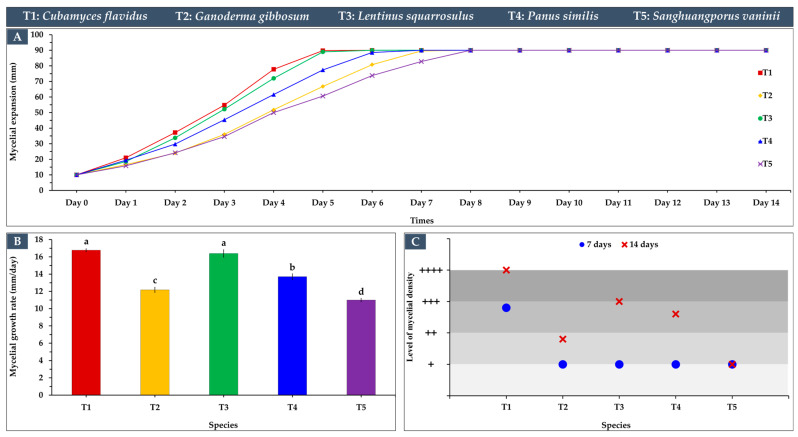
Mycelial growth characteristics of five edible and medicinal mushroom species cultivated on sawdust substrates: (**A**) mycelial expansion patterns, (**B**) growth rate across the incubation period (means ± SD), with statistical differences indicated by letters (a–d) using Duncan’s multiple range test (*p* ≤ 0.05), and (**C**) mycelial density levels.

**Figure 4:**
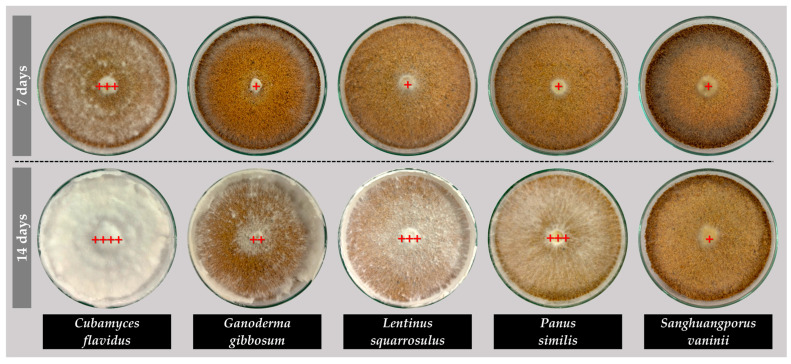
Mycelial density of five edible and medicinal mushroom species grown on sawdust-based substrates. Density level was defined as very thin (+), thin (++), thick (+++), and very thick (++++) and is indicated by red plus marks.

The mycelial growth characteristics of the five edible and medicinal mushroom species cultivated on sawdust substrates revealed notable differences in growth rates, density, and expansion patterns (Figure 3), providing considerable guidelines for developing leather-like materials. *Cubamyces flavidus* and *L. squarrosulus* exhibited the highest growth rates, 16.77 mm/day and 16.39 mm/day, respectively (Figure 3B), exhibiting full plate colonization around day 5 (Figure 3A), demonstrating rapid substrate colonization and high potential for producing dense mycelial mats (Figure 3C). Their mycelial density was also high, particularly for *C. flavidus*, which achieved a rating of +++ at day 7 and ++++ at day 14 (Figure 4), indicating an excellent and tightly interwoven hyphal network suitable for forming leather-like textures. *Panus similis* displayed moderate growth (13.70 mm/day), achieving complete expansion by day 6–7, while *G. gibbosum* and *S. vaninii* showed slower growth rates (12.17 mm/day and 11.00 mm/day, respectively), requiring until day 8 for full plate coverage. Despite slower rates, these species still formed dense mycelial networks over time, suggesting potential for applications requiring thicker or more resilient material structures.

The expansion patterns demonstrated that rapid early growth, as seen in *C. flavidus* and *L. squarrosulus*, contributed to uniform coverage and denser mycelial mats, which are critical factors in achieving leather-like texture, durability, and reducing the risk of contamination [12]. Conversely, slower-growing species such as *S. vaninii* and *G. gibbosum* may require longer cultivation periods or optimization of substrate composition to reach comparable density and uniformity. These differences highlight the fact that mycelial growth characteristics vary widely among species, influenced by critical factors such as culture type, substrate composition, and ambient environmental conditions [32].

These results suggest that species selection and management of mycelial growth parameters are key to producing sustainable, eco-friendly leather-like materials, aligning with current trends in green fashion by utilizing renewable fungal biomass. Overall, the study demonstrates that edible and medicinal mushrooms possess diverse growth characteristics that can be strategically harnessed to develop biodegradable and high-performance fungal materials for long-term uses.

### 3.2. Physical Properties and Characteristics

#### 3.2.1. Visual Characteristics

The visual assessment of mushroom mycelium skin revealed distinct differences in color, texture, and surface uniformity among the species (Table 1 and Figure 2), which are critical for evaluating their potential as mycelium-based material products for sustainable design applications [33]. *Cubamyces flavidus* exhibited a range of whitish to yellowish tones, from pale yellow to light orange, with a predominantly smooth surface interrupted by scattered primordia-like bumps. This combination of light coloration and subtle texture variation suggests potential for applications where a delicate, natural appearance is required. On the other hand, *G. gibbosum* exhibited a broader color spectrum, ranging from whitish and orange-white to reddish golden, brown, and deep brown shades. Its surface was mostly rough with noticeably coarse areas, indicating a more rugged and textured appearance that could mimic certain vintage leather styles but may require post-processing for uniformity. *Lentinus squarrosulus* produced sheets with a consistent rough and hard texture, spanning whitish and pale orange to dark brown and brownish gray. The uniform roughness and dense appearance suggest strong structural integrity but a less pliable feel compared to smoother mycelium sheets, which might make it as suitable for applications where durability is prioritized over softness. *Panus similis* showed a smooth, largely uniform surface across a gradient from whitish and orange-white to rich brown shades, with only minor rough areas. Its balance of smoothness and visual depth makes it an achievable possibility for high-quality mycelium-like leather with both esthetic appeal and functional flexibility. Interestingly, *S. vaninii* demonstrated the smooth and most consistent surface among the mushroom mycelium sheets, with only minor variations in texture. Its uniform appearance and refined tactile quality closely resemble conventional leather, suggesting its possibilities for exclusive, decorative uses.

For comparison, cow tanned leather displayed a warm brownish orange palette with a consistently smooth surface, which reflected the beautiful look of actual leather products [40]. PU microfiber leather exhibited deep chocolate brown tones with an exceptionally smooth and uniform surface, typical of synthetic leathers designed for a signature look [41].

These observations suggest that mycelium-like leather sheets, particularly those from *P. similis* and *S. vaninii*, can achieve primary visual and textural qualities similar to traditional leather. Variations in color and roughness across species may offer opportunities of personalizing feeling and esthetic characteristics for various design uses. The ability to balance smoothness, texture, and natural coloration supports the feasibility of using mushroom mycelium as a sustainable, eco-friendly alternative to conventional leather, aligning with contemporary trends in environmentally conscious design.

#### 3.2.2. Shrinkage and Density

The physical evaluation of mycelium-like leathers revealed notable differences in shrinkage and density among the species studied (Table 2), as well as in comparison to traditional cow tanned and PU microfiber leathers. Shrinkage values varied significantly, with *C. flavidus* and *P. similis* exhibiting the highest shrinkage rates at 24.09% and 22.68%, respectively, while *G. gibbosum* displayed the lowest among the mycelium species at 13.17%. *Sanghuangporus vaninii* and *L. squarrosulus* showed average shrinkage values of 18.96% and 21.44%, respectively. Compared to cow tanned leather with shrinkage greater than 5% and PU microfiber leather with minimal shrinkage (0.1–1.2%), the mycelium-based materials generally exhibited a lack of dimensional stability [14,42], suggesting that post-processing stabilization may be needed to improve shape retention.

Apparent density is a key parameter for evaluating materials, as it serves as a reliable predictor of other important properties such as porosity, water absorption, mechanical strength, and thermal properties, making it a valuable indicator of overall mechanical performance [14,43]. Cow tanned leather had the highest density at 0.49 g/cm^3^, reflecting its tightly packed collagen matrix, followed by PU microfiber leather at 0.38 g/cm^3^. Among the mycelium-based leathers, *G. gibbosum* (0.30 g/cm^3^) and *S. vaninii* (0.21 g/cm^3^) were relatively denser, whereas *C. flavidus* (0.13 g/cm^3^) and *P. similis* (0.14 g/cm^3^) exhibited lower densities, indicating more porous, loose hyphal networks. For *L. squarrosulus*, the density was average at 0.17 g/cm^3^. These differences in density correlate with the microstructural observations, where looser hyphal arrangements create lighter, while compact and continuous mycelial networks may improve strength, flexibility, and shape retention [44].

**Table 2:** Overall physical properties of mycelium-like leathers produced from the mycelia of various edible and medicinal mushroom species in comparison to traditional synthetic and animal leathers.

Species/Types	Shrinkage (%)	Thickness (mm)	Weight (g)	Density (g/cm^3^)
T1. *Cubamyces flavidus*	24.09 ± 1.37 ^a^	1.29 ± 0.20 ^c^	0.42 ± 0.08 ^d^	0.13 ± 0.01 ^f^
T2. *Ganoderma gibbosum*	13.17 ± 3.39 ^c^	1.87 ± 0.26 ^b^	1.40 ± 0.18 ^b^	0.30 ± 0.02 ^c^
T3. *Lentinus squarrosulus*	21.44 ± 1.38 ^ab^	1.06 ± 0.05 ^c^	0.44 ± 0.04 ^d^	0.17 ± 0.02 ^e^
T4. *Panus similis*	22.68 ± 1.02 ^a^	0.50 ± 0.04 ^d^	0.17 ± 0.02 ^e^	0.14 ± 0.01 ^f^
T5. *Sanghuangporus vaninii*	18.96 ± 1.61 ^b^	1.76 ± 0.41 ^b^	0.92 ± 0.23 ^c^	0.21 ± 0.03 ^d^
T6. Cow tanned leather	>5 [45]	2.39 ± 0.05 ^a^	2.91 ± 0.12 ^a^	0.49 ± 0.02 ^a^
T7. PU microfiber leather	0.1–1.2 [46]	1.06 ± 0.03 ^c^	1.02 ± 0.02 ^c^	0.38 ± 0.01 ^b^

**Note:** Values are presented as the mean ± standard deviation. Within each species or type of leather, values followed by different letters in the same column are significantly different, as determined by Duncan’s multiple range test (*p* ≤ 0.05).

The combination of shrinkage and density data suggests that the mechanical and dimensional properties of mycelium-like leathers can be tuned by selecting specific fungal species and optimizing growth conditions. Lower-density species such as *C. flavidus* and *P. similis* may offer enhanced softness and breathability, whereas denser species like *G. gibbosum* and *S. vaninii* could provide greater durability and structural integrity. Compared with traditional leathers, mycelium-based materials present a unique balance between lightweight, flexibility, and eco-friendly biodegradability, supporting their potential use in green fashion applications. Post-processing treatments, including controlled drying, pressing, and substrate refinement, could further improve shrinkage control and density uniformity, producing mycelium leathers that meet industry standards for performance and esthetics.

### 3.3. Surface Structures of Mycelium-like Leather Sheets

**Figure 5:**
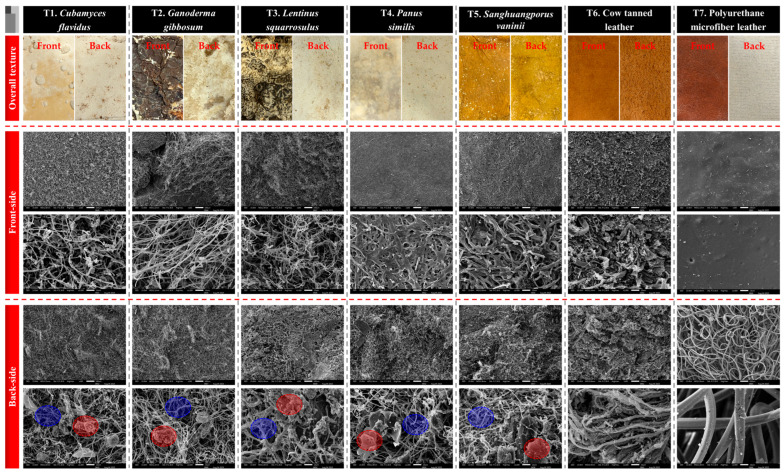
Scanning electron microscope images showing the surface structures of mycelium-like leather sheets derived from the mycelia of five edible and medicinal mushroom species within study, as versus traditional synthetic and animal leathers. The front and back surfaces are presented, with blue highlights indicating areas of pure mycelium, while red highlights mark regions on the backside where sawdust particle residues remain.

SEM offered significant information into the microstructural properties of the mycelium-like leather sheets compared with traditional cow leather and PU microfiber leather (Figure 5). The overall surface textures of the mycelium-derived materials revealed unique natural patterns that closely simulate the textured appearance of animal leather, with some species such as *L. squarrosulus*, *P. similis*, and *S. vaninii* displaying more consistent and compact surface morphology. The front surfaces of the mycelium sheets exhibited a dense network of interwoven hyphae, forming a continuous and cohesive mat. This interconnected hyphal arrangement is essential for imparting mechanical strength, flexibility, and the leather-like feel that is desirable for fashion applications [12,44].

On the backside, SEM images highlighted notable differences among species. Blue-highlighted regions represented areas of pure, well-colonized mycelium, whereas red-highlighted areas indicated residual sawdust particles partially contained within the mycelial network. These residues were more apparent in mycelium sheets than in cow leather or PU leather, suggesting that optimizing substrate composition or improving post-harvest cleaning could enhance surface purity and uniformity.

When compared to cow tanned leather, which displayed a fibrous collagen matrix [47,48], the mycelium sheets appeared simpler and more porous, providing enhanced breathability, and lightweight characteristics [11,49]. PU microfiber leather, on the other hand, exhibited a highly uniform and synthetic surface lacking the natural fibrous microstructure, pores, and smell found in both cow leather or mycelium sheets [50]. This structural difference highlights the inherent advantage of mycelium-based materials in offering a natural, biodegradable alternative with a microstructure that supports both physical comfort and environmental compatibility.

All things considered, the SEM results demonstrate that mycelium-like leathers can potentially approach the hierarchical structure of conventional leathers while providing unique opportunities to modify texture, porosity, and physical characteristics by carefully selecting the appropriate substrate and growth conditions. The presence of sawdust residues on the back surface may be further minimized through substrate refinement or surface finishing treatments, leading to a more consistent product that aligns with the expectations of eco-conscious fashion industries.

### 3.4. Water Absorption

**Figure 6:**
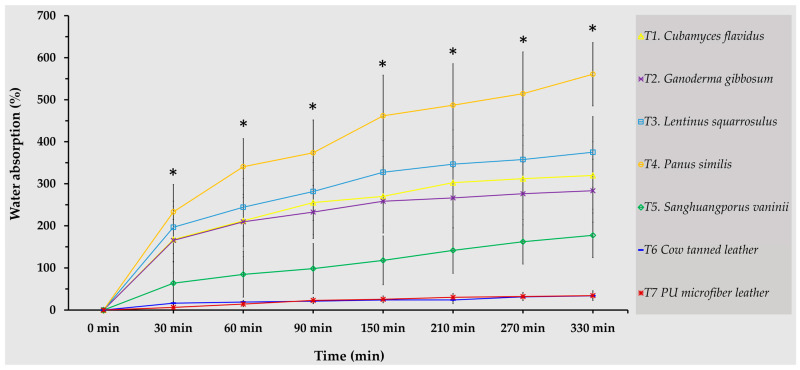
Water absorption behaviors of mycelium-like leather sheets compared with conventional leathers. Data are presented as the mean values, with error bars representing ± standard deviation. Asterisks (*) indicate statistically significant differences at each point, as determined by Duncan’s multiple range test (*p* ≤ 0.05).

The water absorption profiles of the mycelium-like leather sheets and conventional leathers over a 330 min period are presented in Figure 6. Distinct patterns were observed between the mycelium-based samples and the conventional controls (cow tanned leather and PU microfiber leather). Most of the mycelium-based samples exhibited rapid water uptake within the first 90 min, followed by a gradual stabilization phase. Among the tested species, *P. similis* demonstrated the highest water absorption capacity, reaching 560.67% at 330 min, whereas *S. vaninii* absorbed considerably less, with a final value of 177.26%. Notably, *L. squarrosulus* showed a balanced absorption profile, achieving 375.18% at 330 min, suggesting a dense yet moderately porous mycelial network. *C. flavidus* and *G. gibbosum* followed similar patterns, stabilizing at 319.77% and 283.43%, respectively. In contrast, the conventional leather controls exhibited minimal water uptake. PU microfiber leather absorbed only 34.22%, while cow tanned leather showed the lowest overall absorption (33.74%), indicating strong resistance to water penetration compared with the mycelium-like leathers.

The differences in water absorption among fungal species can be attributed to variations in overall density, microporous structure, and the characteristics of the outer hydrophobic layer. Mycelium with higher density and more compact hyphal networks tends to exhibit lower water absorption, whereas more porous structures allow greater moisture uptake [51,52]. Overall, the results clearly demonstrate that mycelium-based leather sheets exhibit markedly higher water absorption than conventional animal-derived and synthetic leathers. This tendency arises from the characteristic porosity and water-attracting nature of fungal mycelia, which form capillary-like networks that easily get and retain moisture [53,54]. For example, *P. similis*, *L. squarrosulus*, and *C. flavidus* likely develop looser overall mycelial networks (both front and backside) with larger capillary spaces, leading to greater water uptake, while *S. vaninii* forms denser, more compact structures that provide better resistance to moisture. These findings align with previous studies, which highlight that the porous structure of mycelial skin and its biochemical composition play key roles in influencing mechanical strength, structural characteristics, as well as water barrier performance [14,54,55]. Notably, water absorption in mycelium-based leathers and pure mycelium materials generally falls within the range of 53.08–496% [47,56,57], highlighting both their potential and the need for further material optimization.

From a sustainability perspective, while the high-water absorption of mycelium leathers poses a challenge for practical use in fashion applications, it also provides opportunities for targeted material engineering. Strategies such as surface modification, blending with hydrophobic biopolymers, or incorporating natural waxes could significantly reduce water uptake while maintaining eco-friendly attributes. Importantly, the data suggest that selective species choice, favoring naturally lower-absorption fungi like *S. vaninii*, may already offer a viable starting point for developing water-resistant mycelium leathers without extensive modification. All things considered, mycelium-like leathers offer beneficial structural and sustainable advantages, though controlling water absorption is essential to following conventional leather standards in eco-friendly fashion.

### 3.5. Thermogravimetric Analysis

**Figure 7:**
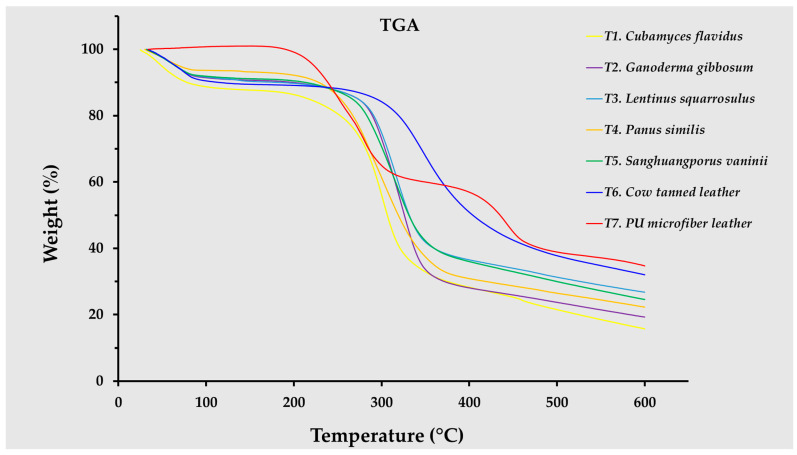
Thermogravimetric analysis of mycelium-like leathers derived from the mycelia of five edible and medicinal mushroom species investigated in this study, compared with conventional synthetic and animal leathers.

TGA revealed distinct thermal degradation profiles for mycelium-like leather materials compared with conventional leathers (Figure 7), offering critical details into their thermal stability and potential application in sustainable fashion. All mycelium-derived samples (*C. flavidus*, *G*. *gibbosum*, *L*. *squarrosulus*, *P*. *similis*, and *S*. *vaninii*) exhibited a three-stage weight loss pattern: an initial minor mass loss below 150 °C associated with moisture evaporation, followed by a major decomposition phase between 200 and 400 °C, and a final slow degradation phase above 450 °C linked to the breakdown of chitin, carbohydrates, amino acids, and other cell-wall components [58]. Among the fungal species tested, *S. vaninii* and *G. gibbosum* demonstrated slightly higher thermal stability, retaining more than 35% of their mass at 400 °C, suggesting a denser, more thermally resistant network structure.

When compared with cow tanned leather and PU microfiber leather, mycelium-like leather sheets exhibited comparable or even superior thermal degradation behavior in the mid-temperature range (250–400 °C), with some species such as *L. squarrosulus* closely replicating the thermal profile of animal leather. Notably, PU microfiber leather showed a delayed onset of weight loss but a more gradual and extended degradation curve, reflecting its synthetic polymer composition. This property might provide better dimensional stability at moderate temperatures but raises concerns regarding recyclability and end-of-life environmental impact due to persistent polymer residues.

These results collectively indicate that mycelium-like leathers offer a combination between biodegradability and thermal stability that may be appropriate for use in designs. Their thermal performance is robust enough to withstand conventional product manufacturing processes such as hot pressing, stamping, and heavy steaming, while still aligning with the sustainability goals of biodegradability and lower carbon footprint. Optimizing substrate formulations and growth conditions could further enhance the thermal resistance of these biomaterials, bringing them even closer to, or surpassing, the performance of traditional leather products while offering a more circular and eco-friendlier alternative.

### 3.6. Further Activities and Future Perspectives

To advance the development of mushroom mycelium as a viable leather-like material aligned with eco-friendly and sustainable fashion trends, several future directions are proposed. A promising approach involves reinforcing mycelium with natural fibers such as cheesecloth, woven fabrics, and other biodegradable materials to improve structural strength, flexibility, and surface texture [12,59], offering a composite solution that remains biodegradable and sustainable.

In addition, the adoption of eco-friendly tanning methods presents a valuable opportunity to improve material performance while avoiding harmful chemicals used in conventional leather processing. Techniques such as vegetable tanning and enzymatic treatments (e.g., microbial proteases) can increase the leather’s softness, flexibility, and uniformity, while preserving its natural feel, without the need for substances like chrome, glycerol, ethylene glycol, or synthetic tanning oils [12,23,60].

Surface coating also plays a vital role in enhancing the performance, functionality, and esthetic appeal of mycelium leather. Biodegradable coating agents, like natural dyes, resins, oils, bio-paraffins, polylactic acid, and bio-based polymers, can be applied using techniques like air spraying, curtain coating, or dip coating. These coatings improve water resistance, color stability, and surface finish while staying true to the principles of sustainability [7,15,57]. Future research should also explore how mycelium leather responds to washing, including its resistance to wear, retention of color, and stability of texture, as well as its interactions with different detergents. Understanding these factors is essential for evaluating the durability and care requirements of fashion products made from this material. The determination of cleaning agents is generally equally important. For instance, strong or alcohol-based cleansers should be avoided since they may dry up or fade the material. Therefore, comfortable, pH-balanced solutions with natural ingredients should be used [61]. However, one key limitation that remains underexplored is the long-term stabilization and aging behavior of mycelium-like leather under various environmental conditions. Over time, environmental factors such as humidity and sunlight exposure could influence the material’s flexibility, color, and structural integrity. Investigating these aging mechanisms and developing stabilization strategies, such as natural cross-linking or eco-friendly maintaining treatments, will be essential to maximize the material’s long-term use and market acceptance.

**Figure 8:**
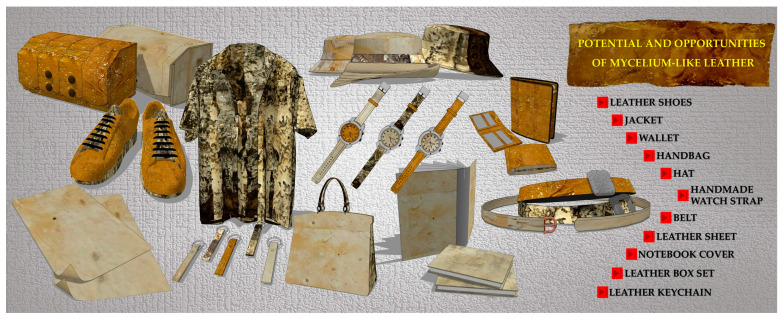
Future directions and opportunities for incorporating mycelium-like leather innovations into the creation of stylish and completely biodegradable prototypes.

Looking ahead, scaling up production through an adjusted process, particularly by increasing the size and length of mushroom spawns, will available the generation of larger mycelium-like leather sheets. These innovations will be integrated into the design and production of stylish, fully biodegradable prototypes, such as shoes, wallets, jackets, handbags, hats, handmade watch straps, belts, notebook covers, leather box sets, keychains, sheets, and other fashion accessories and garments (Figure 8). These next steps are essential to validating the material’s real-world performance, design versatility, and consumer appeal. Ultimately, this approach supports the change toward a circular fashion economy, reducing the environmental footprint of synthetic leathers and decreasing reliance on traditional animal-based products.

## 4. Conclusions

This study demonstrates the promising potential of mushroom mycelium and provides a comparative analysis of five species cultivated on sawdust substrates as a sustainable alternative for developing leather-like materials aligned with eco-friendly fashion trends. Among the five species evaluated, *C. flavidus* and *L. squarrosulus* exhibited the fastest mycelial growth rates, achieving full substrate colonization by day 5 and forming dense, tightly interwoven hyphal networks. Such rapid growth is critical for producing consistent, strong mats suitable for leather-like applications. Species with slower growth, including *G. gibbosum* and *S. vaninii*, required longer cultivation periods but still developed compact structures, suggesting their suitability for products requiring thicker or more durable materials. Visual and microstructural analyses indicated that *P. similis* and *S. vaninii* produced the smoothest and most uniform surfaces, closely resembling conventional cow leather, whereas *C. flavidus*, *G. gibbosum*, and *L. squarrosulus* offered unique textural features that might expand the range of possibilities. Shrinkage and density measurements demonstrated the adaptable physical properties of mycelium-like leathers: *C. flavidus* and *P. similis* exhibited higher shrinkage with lower densities, favoring softness and airflow, whereas denser species such as *G. gibbosum* and *S. vaninii* offered greater structural integrity. TGA revealed robust stability, with *S. vaninii* and *G. gibbosum* retaining over 35% mass at 400 °C, indicating their capacity to withstand conventional manufacturing processes while remaining biodegradable. Water absorption studies further illustrated species-specific behavior, with *P. similis* exhibiting the highest uptake and *S. vaninii* the lowest, demonstrating the significance of structural density in moisture resistance. Collectively, these findings highlight that mycelium-like leathers can combine the desirable mechanical strength, flexibility, and esthetic appeal with environmentally sustainable attributes. Future optimization through substrate refinement, eco-friendly tanning, surface coatings, and fiber reinforcement can enhance performance, water resistance, and uniformity. With these advancements, mycelium holds strong potential as a renewable and biodegradable alternative to animal and synthetic leather, offering opportunities to support the circular economy, minimize environmental impact, and drive innovation in sustainable fashion.

## Figures and Tables

**Table 1:** Initial characteristics and classification of mushroom mycelia to determine the best possible species for high-quality mycelium-like leather.

Species/Types	Sheet Characteristics
T1. *Cubamyces flavidus*	**Type of mushroom:** Medicinal **Decay type of mushroom:** White rot **Hyphal system:** Trimitic **Color and texture:** Whitish to Yellowish Shades: Ranging from mostly and yellowish white to light yellow (4A2–4), often grayish yellow (4B4–6), and somewhere pale to light orange (5A3–4). The surface is mostly smooth, though scattered primordia-like bumps provide slight inconsistencies in texture.
T2. *Ganoderma gibbosum*	**Type of mushroom:** Medicinal **Decay type of mushroom:** White rot **Hyphal system:** Trimitic **Color and texture:** Warm Orange to Deep Brown Tones: Various in color from whitish and orange-white hues, progressing to light orange (5A2–5), reddish golden to brownish orange (6C3–4), light brown (6D4–5) to brown (7E4–8), and even reddish to dark brown (8F4–8). The surface is mostly rough, with certain distinctly coarse areas.
T3. *Lentinus squarrosulus*	**Type of mushroom:** Edible **Decay type of mushroom:** White rot **Hyphal system:** Dimitic **Color and texture:** Orange-White to Dark Earthy Colors: Ranging from whitish and pale orange (5A2–3) to brown (7E3–7), dark brown (7F5–8), and brownish gray (7C2). The entire sheet has a consistently rough, hard texture that gives it a rugged appearance.
T4. *Panus similis*	**Type of mushroom:** Medicinal **Decay type of mushroom:** White rot **Hyphal system:** Dimitic **Color and texture:** Orange-White to Rich Brown Gradient: Featuring whitish, orange-white, light orange (5A2–4), grayish orange (5B3–4, 6B3–5), light brown (6D4–5), and brown (6E6). The sheet’s surface is largely smooth and uniform, with only minor roughness in small areas.
T5. *Sanghuangporus vaninii*	**Type of mushroom:** Medicinal **Decay type of mushroom:** White rot **Hyphal system:** Dimitic **Color and texture:** Light brown (6D5–8), brown to dark brown (6F7–8). The surface is smooth throughout, with a few slightly rough sections giving some textural variation.
T6. Cow tanned leather	**Color and texture:** Warm Brownish Orange Palette: Dominated by brownish orange (6C8) and deep brown (6D8). The surface is consistently smooth, giving it a refined, cleaned appearance.
T7. Polyurethane microfiber leather	**Color and texture:** Deep Chocolate Browns: Transitioning from brown (7E8) to dark brown (8E7–8). The sheet exhibits an exceptionally smooth and uniform surface, providing it a refined, polished look.

**Note:** Key references informing this study include Kornerup and Wanscher [24], Aiduang et al. [28], Srinivasarao and Nagadesi [34], Luangharn et al. [35], Ishaq et al. [36], Yue et al. [37], Shin [38], and Balan et al. [39].

## Data Availability

The original contributions presented in this study are included in the article. Further inquiries can be directed to the corresponding author.
